# Caspase-Like Proteases as Contributors in the Functioning of Reproductive Barriers During Distant Hybridization in Solanaceae

**DOI:** 10.3390/plants15193048

**Published:** 2026-10-06

**Authors:** Alexej I. Ulianov, Yaroslav Yu. Golivanov, Tatiana P. Molchanova, Oksana A. Muratova, Natalia A. Milyukova, Ekaterina V. Zakharova

**Affiliations:** All-Russia Research Institute of Agricultural Biotechnology, 127550 Moscow, Russia

**Keywords:** distant hybridization, intergeneric reproductive barriers, self-incompatibility, caspase-like proteases, Solanaceae

## Abstract

Distant hybridization is an important breeding strategy that can effectively expand the genetic pool for developing new, high-yielding, and stress-resistant cultivars. However, reproductive isolation often hinders the application of this approach. Intergeneric reproductive barriers (IRBs) manifest during pollen–pistil interactions in plants belonging to different genera, yet the mechanisms underlying these barriers remain largely unknown. In this study, we investigated pollen–pistil interactions during the progamic phase of fertilization in three Solanaceae species using several intergeneric cross combinations. Pollen of all tested species germinated on the stigma surface, grew through the stylar transmitting tissue, and ceased elongation at a distance of 300–920 µm therefrom due to the action of a reproductive barrier. We suggest that programmed cell death (PCD) is a factor contributing to IRBs in Solanaceae. The obtained data indicate that, in all cross combinations, the rejection of incompatible pollen tubes (PTs) is accompanied by increased activity of caspase-like proteases (CLPs), as confirmed by in vivo visualization of active CLPs and spectrophotometric measurement of their activity. Specifically, incompatible PT growth in stylar tissues during the first 4 h after pollination was accompanied by a five- to six-fold increase in caspase-3-like protease activity. Notably, the arrest of PT growth in distant hybridization shows striking similarity to that observed during S-RNase-based self-incompatibility (SI) in Solanaceae. We anticipate that further research will ultimately provide insights into the regulation of reproductive barriers during distant hybridization.

## 1. Introduction

Distant hybridization, involving gene exchange between different genera and species, plays a crucial role in crop improvement [1,2]. Many cultivated crops, including rice [3,4,5,6], wheat [7,8,9], and crucifers [10,11], have been developed through distant hybridization. However, this process is often challenging or even impossible due to interspecific/intergeneric reproductive barriers (IRBs), which prevent successful fertilization and hybrid embryo development, thereby representing a significant bottleneck in breeding and limiting the exploitation of valuable gene pools [12].

The transition to highly productive and environmentally sustainable agriculture requires expanding the diversity of cultivars and hybrids of major crops, along with increasing yield, improving product quality, and enhancing plant tolerance to biotic and abiotic stresses [13,14]. Traditional breeding methods are increasingly insufficient to achieve these goals because the genetic resources available for conventional breeding are limited, as successful crosses are generally restricted to plants within the same biological species or between very closely related species [15]. Meanwhile, representatives of other genera within the same family often possess agronomically valuable traits, particularly resistance to pathogens and abiotic factors, which are of considerable interest for breeding [16]. Therefore, a comprehensive investigation of IRBs is important not only from a fundamental perspective but also for providing a basis for future breeding efforts aimed at overcoming these barriers.

Current understanding of pollen–pistil interactions underlying IRBs is very limited, and the associated physiological and molecular mechanisms remain largely unexplored. Solanaceae species, including *Petunia hybrida* E. Vilm. and *Nicotiana tabacum* L., which we have studied extensively, serve as convenient models for investigating the mechanisms of intergeneric incompatibility.

In recent years, our group has demonstrated that programmed cell death (PCD) is a factor in gametophytic self-incompatibility (SI) in *P. hybrida* and in self-incompatible wild tomato species such as *Solanum chilense* Reiche., *Solanum habrochaites* S. Knapp & D.M. Spooner, and *Solanum pennellii* Correll [17,18]. Hormonal signals and their receptors govern intercellular interactions, playing a critical role in regulating pollen tube (PT) growth during both compatible and self-incompatible pollinations [18]. The arrest of self-incompatible PT growth is associated with elevated activity of caspase-like proteases (CLPs) within the first hours after pollination, and by the time growth ceases, all hallmarks of PCD are evident [19]. By treating petunia and tomato stigmas with the caspase-3/DEVDase inhibitor Ac-DEVD-CHO, we successfully overcome SI, resulting in fertilization and the production of viable seeds [17,20]. Based on these findings, we hypothesized that similar processes and mechanisms underlying PT growth arrest and subsequent death may operate during intergeneric incompatibility in Solanaceae, and that understanding these mechanisms may ultimately enable successful intergeneric hybridization.

Overall, the primary objective of this study was to investigate whether caspase-like proteases are involved in pollen tube growth arrest during intergeneric incompatibility in Solanaceae, and to compare this process with S-RNase-mediated self-incompatibility.

While our previous studies described the phenomenological similarity between SI and IRB, the present work provides the first molecular evidence that CLPs are activated during intergeneric incompatibility.

## 2. Results

### 2.1. Dynamics of Pollen Grain Germination and Pollen Tube Growth In Vivo During Distant Hybridization

Visualization of growing PTs in vivo revealed that in all intergeneric cross combinations, the majority of tobacco, salpiglossis, or petunia pollen grains (PG) germinated on the stigma surface, grew through the stylar transmitting tissue, and ceased elongation approximately 6 h after pollination (Figure 1, Table 1). At 2 h after pollination, the mean PT length in the SC × N combination was approximately 400 µm; it reached about 700 µm at 4 h and 920 µm at 6 h, and remained at approximately 920 µm until 24 h after pollination. No seed set was observed. A similar pattern was observed for the SC × SS hybridization at all time points. PT behavior differed when pistils of self-incompatible petunia were pollinated. In both intergeneric cross combinations involving SI pistils (SI × N and SI × SS), PTs reached a length approximately three times less than that observed in crosses with SC petunia pistils. By 6 h after pollination, PT length reached approximately 300 µm (Figure 1, Table 1).

The overall pattern of PT germination and growth under IRBs resembles that observed during SI. In petunia SI, PTs arrest their growth at a distance of approximately 8 mm from the stigma surface at 6–8 h after pollination [21]. PTs in distant hybridization did not reach this length; their final length varied depending on the cross combination (Table 1), and the time of growth arrest was observed at 6 h after pollination (SC × N, SI × SS).

Treatment of petunia pistils with the caspase-3 inhibitor (0.25 mM) resulted in a significant increase in the length of both tobacco (*N. tabacum*, N) and salpiglossis (*S. sinuata*, SS) PTs growing in petunia pistil tissues (Table 1). Two-way ANOVA revealed a significant effect of the inhibitor on PT length at all investigated time points for all combinations (*p*-values ranged from < 0.001 to < 0.05; Tukey’s post hoc test).

At 4 h after pollination, PT length in the presence of the inhibitor was 1141 ± 31 µm (SC × N) and 951 ± 35 µm (SC × SS), which was significantly higher than the corresponding control values (688 ± 88 and 484 ± 36 µm, respectively; *p* < 0.001).

The final PT length after growth arrest was also significantly increased: 1431 ± 129 µm (SC × N, 24 h), 1423 ± 41 µm (SI × N, 24 h), 1216 ± 55 µm (SC × SS, 24 h), and 1069 ± 34 µm (SI × SS, 24 h) (*p* < 0.001 for all variants; Tukey’s post hoc test).

The greatest relative increase in PT length under inhibitor treatment was observed in crosses involving the self-incompatible petunia line (SI × N and SI × SS), with a 3.7-fold and 3.4-fold increase compared to the control, respectively, whereas in self-compatible variants, the increase was 1.4-fold (SC × N) and 1.2-fold (SC × SS). Two-way ANOVA for the 24 h time point revealed a significant interaction between the factors “inhibitor” and “cross combination”, confirming the differential efficacy of the inhibitor depending on the cross.

Inhibitor treatment did not alter the timing of growth arrest: in the SI × SS variant, growth ceased by 6 h (mean PT lengths at 6 h and 24 h were not significantly different: 1068 ± 36 vs. 1069 ± 34 µm; *p* = 0.94, *t*-test), whereas in SC × N, SI × N, and SC × SS variants, PTs continued to grow significantly up to 24 h (*p* < 0.05 for all three variants, *t*-test). No seed set was observed in any of the inhibitor-treated variants.

Thus, in contrast to SI, where treatment with the caspase-3 inhibitor completely overcomes the barrier and results in seed formation [17], the inhibitor exerts only a partial effect during intergeneric hybridization—it increases PT length but does not fully overcome the reproductive barrier in this experiment. Further optimization of the inhibitor concentration and treatment timing may be required to achieve complete suppression of PCD during intergeneric hybridization.

### 2.2. Activity of CLPs in the Pollen–Pistil System During Intergeneric Hybridization

To determine caspase-3-like protease activity in the pollen–pistil system across different pollination variants, we employed the fluorogenic caspase-3 substrate Ac-DEVD-AMC. This substrate is cleaved by activated DEVD-specific proteases, releasing a fluorescent product that allows activity measurement. Caspase-3-like protease activity in the pollen–pistil system varied depending on the type of pollination (Figure 2). In the case of cross-compatible pollination (SC × SC), we observed a consistently low level of CLP activity in pistil tissues, which was accompanied by unidirectional growth of compatible PTs through the stylar tissues over a 24 h period.

In contrast, the pattern of caspase-3-like protease activity during self-incompatible pollination was markedly different. Germination and growth of self-incompatible PTs in the stigma and style during the first 2 h were accompanied by a sharp increase in caspase-3-like protease activity, followed by a sharp decline over the next 2 h. The pattern of caspase-3-like protease activity during intergeneric hybridization was similar to that observed during self-incompatible pollination. Quantitative differences were evident in the self-compatible *P. hybrida* × *N. tabacum* cross, but the overall dynamics were preserved. A sharp decrease in CLP activity in the pollen–pistil system, both in self-incompatible pollination and in distant hybridization, was observed at 6 h after pollination, coinciding with the arrest of PT growth.

The significance of differences between variants in the spectrophotometric assessment of caspase-3-like protease activity was determined using Fisher’s least significant difference (LSD_0.05_) calculated for each time interval. The LSD_0.05_ values were 51.1 (2 h), 51.8 (4 h), 27.0 (6 h), 22.9 (24 h), and 16.7 (48 h). Overall, the highest CLP activity was observed in intergeneric crosses (particularly SC × N), while the lowest activity was consistently found in compatible pollination (SC × SC). At 2 h, enzyme activity in the SC × N and SI × N variants was significantly higher than in SC × SC. At 4 h, the maximum activity was recorded in the SC × N variant, which was significantly higher than all other variants. At 6 h, SC × N also significantly exceeded SI × N, SC × SC, and SI × SI, while differences between SI × N and SI × SI were not significant. By 24 h, activity in SC × N and SC × SC did not differ, but both were significantly higher than SI × SI. At 48 h, maximum activity persisted in the SC × N variant, whereas SC × SC exhibited the lowest activity.

### 2.3. Visualization of Intergeneric-Incompatibility-Induced CLP Activity in Growing Pollen Tubes In Vivo

CLP visualization experimentally demonstrated that the observed CLP activity in the petunia pollen–pistil system during distant hybridization is localized within the PTs, rather than in the pistil cells (Figure 3). The green fluorescent signal within the PTs indicates the presence of active CLPs at the time of detection. Our results suggest that CLP activity is localized in the cytoplasm of PTs, which exhibit a bright signal (Figure 3). CLP activity was visualized in all visible pollen grains and pollen tubes in vivo growing tobacco PTs (Figure 3A) and salpiglossis PTs (Figure 3B) within petunia pistil tissues at 2 h after pollination.

## 3. Discussion

### 3.1. The Pollen–Pistil System

The angiosperm pistil is a unique system (Figure 1). It serves a protective function and acts as a conduit for PTs, enabling them to reach the ovary, while also providing the site for pollen grain (PG)–pistil interactions, regulating PT growth, and hence fertilization [21,22,23]. The stigma surface serves as the receptor site for triggering mechanisms associated with pollination and fertilization. And it is also the initial point for determining incompatibility [24,25].

For successful fertilization, pollen and pistil must be precisely co-adapted. The long journey of the PT through the stigma, style, and toward the micropyle has been comprehensively described in a review [26], where the authors highlighted the precise regulation and molecular dialogue necessary for fertilization. Understanding these interactions, beyond fundamental research, is relevant for controlling the fertilization process.

Extensive histological studies of pistillate flowers have revealed at least four distinct locations of PT arrest during the operation of different incompatibility mechanisms, reflecting unique mechanisms of male gametophyte recognition/selection [27]. The incompatibility reaction can manifest at different stages of pollination and fertilization. Inhibition can occur before or during pollen germination, i.e., on the external surface of the stigma, as observed, for example, in Brassicaceae. PT growth can be inhibited during its progression through the stylar transmitting tissue, as we observe in our studies of SI and IRB in Solanaceae. The site of arrest can also be at the ovule entrance and the incompatibility reaction can even manifest after karyogamy, resulting in the abortion of fertilized egg cells or young embryos [28].

To date, SI mechanisms are the most thoroughly studied pollen–pistil interaction systems at the molecular and genetic levels, allowing their use as a model for investigating pollen rejection processes during interspecific pollination as well [29]. SI serves as an effective reproductive barrier and a mechanism promoting outcrossing between genetically distinct plants, but it also represents a limiting factor when considering plants of agricultural interest [29]. Thus, plants have been able to promote the genetic variability that constitutes an important basis for evolution [27,30].

Our attention is focused on pollen–pistil interactions that contribute to PT selection and intergeneric pollen rejection in Solanaceae plants. The pistil of Solanaceae is wet, covered with a liquid exudate. Viable PGs landing on the stigma surface hydrate, germinate, grow through the stylar transmitting tissue, and arrest their growth within the stylar tissues. We observe this phenomenon both during the operation of the SI mechanism [17,19] and during PT growth arrest under IRBs (Figure 1), which already indicates a similarity between these complex mechanisms.

### 3.2. PCD as a Factor in S-RNase-Based SI in Solanaceae

SI is a genetically controlled process by which many flowering plants prevent self-fertilization and inbreeding [31]. The pollen–pistil interaction in a self-incompatible plant induces a signaling network leading to growth arrest and PCD of incompatible PTs [32]. The Solanaceae-type SI mechanism involves two tightly linked S-locus genes: the S-RNase gene, controlling S-specificity in the pistil, and the S-locus F-box (SLF) gene, controlling S-specificity in pollen [33,34,35,36]. The biochemical mechanism underlying self-pollen tube rejection involves RNA degradation in incompatible PTs. Thus S-RNase functions in SI as an S-specific cytotoxin [37]. S-RNase is synthesized in the stylar transmitting tissue and secreted into the extracellular matrix of the transmitting tract [38].

SI exhibits several hallmarks of PCD, including actin depolymerization, reactive oxygen species signaling, CLP activity, and DNA fragmentation [39]. Although canonical caspase genes have not yet been identified in plant genomes, pro-tease activities with caspase-like properties essential for PCD are observed. CLPs, functional analogs of caspases, participate in plant PCD [40,41,42,43]. Synthetic caspase inhibitors block PCD in both animal and plant systems [44,45]. Peptide caspase sub-strates have been used to detect plant CLP activity since 1998 [46]. The corresponding detected enzymatic activities have been termed DEVDase, VEIDase, YVADase, etc., depending on the specific substrate used [44].

In our previous studies, we demonstrated the presence of PCD markers, such as DNA fragmentation, in in vivo growing self-incompatible PTs of petunia and tomato using various methods, including the TUNEL assay [17,19]. This indicates that PCD is a factor in S-RNase-based SI, leading to growth inhibition and death of self-incompatible PTs 6–8 h after pollination. Disruption of plasma membrane integrity and DNA degradation were observed in these PTs. Transmission electron microscopy reveals complete destruction of PT contents 12 h after self-incompatible pollination. The type of PCD induced by SI in *P. hybrida* remains to be definitively classified. Current classification of plant PCD suggests that the major types are apoptosis-like PCD, senescence, and vacuole-mediated cell death [36,41]. We can only speculate that PCD in self-incompatible PTs closely resembles the apoptosis-like type of PCD, based on the observed membrane integrity disruption, cytoplasmic condensation, and DNA fragmentation.

It has also been shown that PCD in self-incompatible petunia PTs is mediated by CLP activity. CLP activity is detected in 90% of in vivo growing self-incompatible PTs. High CLP activity accompanies the growth of self-incompatible PTs in the style for 2–4 h and coincides with S-RNase activation [47]. Thomas et al. [48] suggested that actin cytoskeleton disorganization precedes the initiation of PCD during in vivo pollen rejection and that *N. alata* S-RNases act during in vivo pollen rejection after subcellular changes have occurred. For instance, actin depolymerization is observed during SI in *Papaver rhoeas* [48], and S-RNase has been shown to induce similar changes in *Pyrus pyrifolia* [49]. Cytokinin may act as a potential activator of CLP activity; at high concentrations, cytokinin can induce cytosolic acidification, a key turning point in CLP activation in self-incompatible PTs during S-RNase-induced PCD [47]. According to our hypothesis, cytokinin, together with CLPs and S-RNase, disorganizes the actin cytoskeleton of self-incompatible PTs, causing loss of membrane integrity, organelle destruction, and subsequent DNA degradation as the final stage of S-RNase-induced SI PCD in *P. hybrida*.

### 3.3. PCD as a Factor in IRB in Solanaceae

Relatively few publications address the similarities and differences between SI and IRB mechanisms. We recently published a comprehensive review on this topic, aiming to highlight aspects related to these questions [50].

The search for evidence of a universal incompatibility mechanism represents a promising direction, as it is unlikely that each species existing in nature has evolved entirely unique mechanisms [51]. Consequently, it is reasonable to expect that incompatibility may be governed by a universal mechanism. At the same time, researchers [52,53,54] are likely correct that a unifying theory of incompatibility will be based not on a single-locus but on polygenic control of the PT growth inhibition response.

Some authors [53,55] consider interspecific incompatibility in close connection with SI, based on the analogy of PT growth arrest in distant hybridization and SI. The hypothesis of a universal incompatibility mechanism stems from the existence of specifically evolved fertilization reactions arising from the interaction of similar alleles controlled by the S-gene. However, during interspecific incompatibility, different, unrelated genes interact, as the chance encounter of identical alleles in distant hybridization is a rare event. Nevertheless, both mechanisms, SI and IRB, are directed toward preventing undesirable fertilization: SI against self-pollination and inbreeding, and IRB against hybridization between different species.

It is generally accepted that interspecific incompatibility is a form of post-pollination prezygotic reproductive barrier that leads to the rejection of foreign species’ pollen within pistil tissues [25,56]. There is also a type of interspecific incompatibility frequently found among closely related angiosperm species, characterized by unidirectional pollen acceptance by the stigma in reciprocal crosses—known as asymmetric unilateral incompatibility (UI).

The hypothesis that SI plays a role in interspecific reproductive barriers is based on the SI × SC (self-compatible) rule, which has been described for various taxa exhibiting either gametophytic or sporophytic SI. According to this rule, when a self-incompatible female form is crossed with a heterospecific self-compatible male form, the SI pistil rejects pollen from the SC species. However, the reciprocal cross (SC × SI) results in successful seed formation. This UI phenomenon indicates the absence of an interspecific pollen rejection mechanism in self-compatible species [57]. In this type of UI, known as the SI × SC rule [58], pollen from SI species on pistils of SC species can lead to hybrid seed formation, but the reciprocal cross (pollen from SC species on pistils of SI species) is ineffective. Thus, cases described in the literature indicate that the IRB mechanism can closely resemble the SI mechanism. Nevertheless, this question remains debatable.

According to our data, prezygotic post-pollination reproductive isolation barriers in Solanaceae plants show similarities to the processes occurring during the operation of the SI mechanism in plants of the same family. Pollen tubes germinate on the stigma surface, grow through the stylar transmitting tissue, and cease elongation 6–8 h after pollination (Figure 1, Table 1) [59]. Biochemical analysis of CLP activity and in vivo visualization of CLPs in growing PTs revealed enzyme activation in incompatible pollen tubes during distant hybridization, which suggest the involvement of PCD.

While our previous study [47] described the phenomenological similarity between SI and IRB—specifically, PT growth arrest at 6–8 h after pollination—the present work provides the first molecular evidence of CLP activation during intergeneric incompatibility (Figure 2 and Figure 3). This distinguishes the current study from our earlier publications [51,60] and suggests that CLPs, known mediators of SI-induced PCD, are also involved in IRB. Direct PCD markers (TUNEL assay, DNA laddering, membrane integrity tests, and TEM analysis) were not obtained for the intergeneric crosses in this study. While CLP activation is an established marker of PCD execution, future studies should confirm these findings using additional PCD markers.

#### Comparison of SI and IRB: Similarities and Differences

Although our data reveal several parallels between SI- and IRB-induced pollen tube rejection, these processes are distinct in their biological purpose, genetic control, and evolutionary significance. Below (Table 2) we summarize the key similarities and differences observed in this and previous studies.

## 4. Materials and Methods

### 4.1. Plant Material and Experimental Design

Clonally propagated plants of self-incompatible (SI) and self-compatible (SC) lines of *P. hybrida* E. Vilm. from the laboratory collection were grown at 26 °C with a 16 h photoperiod. The SI and SC lines of *P. hybrida* used in this study have been described previously [17,18,19]. Plants of *N. tabacum* L. and *Salpiglossis sinuata* Ruiz. & Pav. were grown from seeds and brought to flowering under the same conditions. Salpiglossis plants flowered 8–9 weeks after sowing, while tobacco plants flowered approximately 10–12 weeks after sowing [1]. All plants used in the experiments were at the flowering stage, ranging from up to 12 months of age. Flower emasculation was performed one day before the experiment. On the following day, prepared flowers were hand-pollinated with fresh pollen according to the crossing scheme, using 2 mg of pollen per stigma.

The following crosses were performed:

1. Self-compatible *P. hybrida* ♀ × self-compatible *P. hybrida* ♂ (compatible pollination), SC × SC;
2. Self-incompatible *P. hybrida* ♀ × self-incompatible *P. hybrida* ♂ (self-incompatible pollination), SI × SI;
3. Self-compatible *P. hybrida* ♀ × *N. tabacum* ♂ (intergeneric hybridization), SC × N;
4. Self-incompatible *P. hybrida* ♀ × *N. tabacum* ♂ (intergeneric hybridization), SI × N;
5. Self-compatible *P. hybrida* ♀ × *S. sinuata* ♂ (intergeneric hybridization), SC × SS;
6. Self-incompatible *P. hybrida* ♀ × *S. sinuata* ♂ (intergeneric hybridization), SI × SS.

Pollinated pistils were collected at 2, 4, 6, 24, and 48 h after pollination (the time points for sample collection were selected based on our previous studies on self-incompatibility in *P. hybrida* [17,19]), snap-frozen in liquid nitrogen, and stored at −80 °C until analysis of caspase-3-like protease activity. Material for PT length measurements was fixed in acetic alcohol (ethanol:acetic acid, 3:1) for subsequent visualization and measurement of PT length using aniline blue staining. For all pollination variants, a subset of pollinated pistils was left on the plant for seed set control.

### 4.2. Treatment with Caspase-3 Inhibitor (Ac-DEVD-CHO)

Stigmas were treated with the caspase-3 inhibitor Ac-DEVD-CHO (EMD Biosciences and BIOMOL, San Diego, CA, USA) at a concentration of 0.25 mM by applying a 5 µL drop of the solution onto the stigma surface 2 h before pollination. The concentration was selected based on previous experiments on overcoming SI in petunia [17].

### 4.3. In Vivo Measurement of Pollen Tube Length in Pistil Tissues

#### 4.3.1. Aniline Blue Staining

Fixed pistils were macerated in 20% ethanolic KOH solution for 30 min at room temperature, washed twice with distilled water, and stained with aniline blue (a dye binding to PT callose) for 30–40 min. Stained pistils were placed on a glass slide in a drop of glycerol:water (1:1), covered with a coverslip, and gently squashed. Visualization and measurement of PTs within the stylar transmitting tissue were performed using a BiOptic CI-300 inverted microscope (BIOMED Holding, Moscow, Russia).

#### 4.3.2. Feulgen Staining (Schiff Reagent)

Pollinated pistils were fixed in Carnoy’s fluid for 30 min, washed with 80% ethanol, and dehydrated through a descending ethanol series. The specimens were then placed in 1N HCl (1 normal hydrochloric acid, equivalent to 1 mol/L) at room temperature for 3 min, transferred to 1 N HCl preheated to 60 °C for 5 min, and returned to 1 N HCl at room temperature for 2 min. Subsequently, specimens were stained with Schiff’s reagent for 2 h, washed in three changes of sulfurous water (15 min each) and then in distilled water, air-dried, dehydrated through an ascending ethanol series to absolute ethanol, cleared in xylene, and mounted in Canada balsam.

### 4.4. Analysis of CLP Activity in the Pollen–Pistil System

#### 4.4.1. Protein Extraction

Samples for each variant (10 pistils without ovaries, ~100 mg) were immediately frozen in liquid nitrogen and ground to a fine powder using a pre-cooled mortar and pestle. The powder was suspended in 1 mL of ice-cold protein extraction buffer (20% glycerol, 0.1% Triton X-100, 10 mM EDTA, 3 mM dithiothreitol, 2 mM phenylmethylsulfonyl fluoride, and 50 mM sodium acetate, pH 4.5). Samples were centrifuged at 16,000× *g* for 10 min at 4 °C. Protein concentration was determined according to Bramhall et al. [60].

#### 4.4.2. Spectrophotometric Determination of Caspase-3-like Protease Activity

Caspase-3-like protease activity in pistil extracts was measured as previously described [47] by monitoring the cleavage of the fluorogenic caspase substrate Ac-DEVD-AMC (Ac-Asp-Glu-Val-Asp-AMC; AMC, 7-amino-4-methylcoumarin; EMD Biosciences and BIOMOL, USA). Briefly, 50 µL of sample and 50 µL of 400 µM Ac-DEVD-AMC were added to wells of a 96-well assay plate (Costar, Ithaca, NY, USA,). Fluorophore release upon cleavage was recorded (excitation at 360 nm, emission at 460 nm) using a BioTek Synergy H1 microplate reader (BioTek Instruments, Santa Clara, CA, USA) with a multimode detector. AMC fluorescence was monitored for ~3 h at 10 min intervals. Enzyme activity was calculated using the slope of product concentration as a function of time.

#### 4.4.3. In Vivo Fluorescence Visualization of CLP Activity in Growing Pollen Tubes

CLP activity in living PTs was visualized using the Image-iT™ LIVE Green Poly Caspases Detection Kit (Invitrogen, Thermo Fisher Scientific, Carlsbad, CA, USA). Pistils were collected 2 h after pollination according to the crossing schemes, longitudinally sectioned, and processed following the manufacturer’s protocol. Sections were placed in 1.5 mL tubes, immediately immersed in working solution containing the FLICA reagent (excitation/emission maxima 488/520 nm) in PBS (pH 7.4), using PBS tablets (Helicon, Moscow, Russia), and incubated at room temperature in the dark for 60 min. Sections were then removed from the FLICA reagent, immersed in 5 mM propidium iodide (excitation/emission maxima 490/635 nm), incubated at room temperature in the dark for 10 min, washed twice with wash buffer, mounted on a microscope slide in a drop of wash buffer, covered with a coverslip, gently squashed, and examined immediately. The green fluorescent signal directly indicates the presence of CLP activity at the time of reagent addition.

Images of pistil sections were acquired using an Axio Scope A1 fluorescence microscope (Carl Zeiss, Oberkochen, Germany) equipped with 488/530 nm (for FLICA, green fluorescence) and 535/617 nm (for propidium iodide, red fluorescence) filter sets. Photographs were taken with an AxioCam MRm Zeiss camera (Carl Zeiss, Germany), and contrast was adjusted using ZEN lite Digital Imaging software (ZEISS ZEN lite). For each pollination variant, five individual pistils were stained and imaged. In each experimental variant, the number of PTs analyzed exceeded 200.

### 4.5. Statistical Analysis

Significant differences between cross combinations upon treatment of petunia stigmas with the caspase-3 inhibitor were assessed using two-way analysis of variance (ANOVA) followed by pairwise comparisons of all variants using Tukey’s post hoc test. For spectrophotometric evaluation of caspase-3-like protease activity, the significance of differences between means was assessed using Fisher’s least significant difference (LSD_0.05_) calculated for each time interval. For comparisons of two time points within a single variant, an independent samples *t*-test (Welch’s test) was used. Statistical analyses were performed in the R environment (RStudio 2026.05.1+225 i).

## 5. Conclusions

In this study, we have demonstrated that CLPs play a pivotal role in growth arrest of incompatible pollen tubes during intergeneric hybridization in Solanaceae plants. Caspase-3-like protease is activated in incompatible PTs within the first 2 h of in vivo growth in pistil tissues, and its high activity accompanies PT growth until its cessation at 6–8 h after pollination. In vivo visualization of CLPs confirms that their activation occurs specifically within PGs and PTs.

Furthermore, our results provide preliminary evidence supporting the hypothesis that PT death during the operation of IRB and SI mechanisms follows a similar scenario. We observed comparable dynamics of CLP activity during PT growth and arrest under both IRB and SI conditions. Although we were unable to completely inhibit PCD by treatment with the caspase-3-like protease inhibitor (in contrast to SI in petunia), PT length was significantly increased, and this line of investigation warrants continuation. Studying this problem enhances our understanding of the underlying mechanisms and provides a foundation for future studies aimed at understanding and potentially overcoming reproductive barriers during distant hybridization. Future studies should identify the specific CLP genes involved in IRB, characterize the upstream signals (ROS, calcium, hormones), and analyze downstream targets of PCD execution.

## Figures and Tables

**Figure 1:**
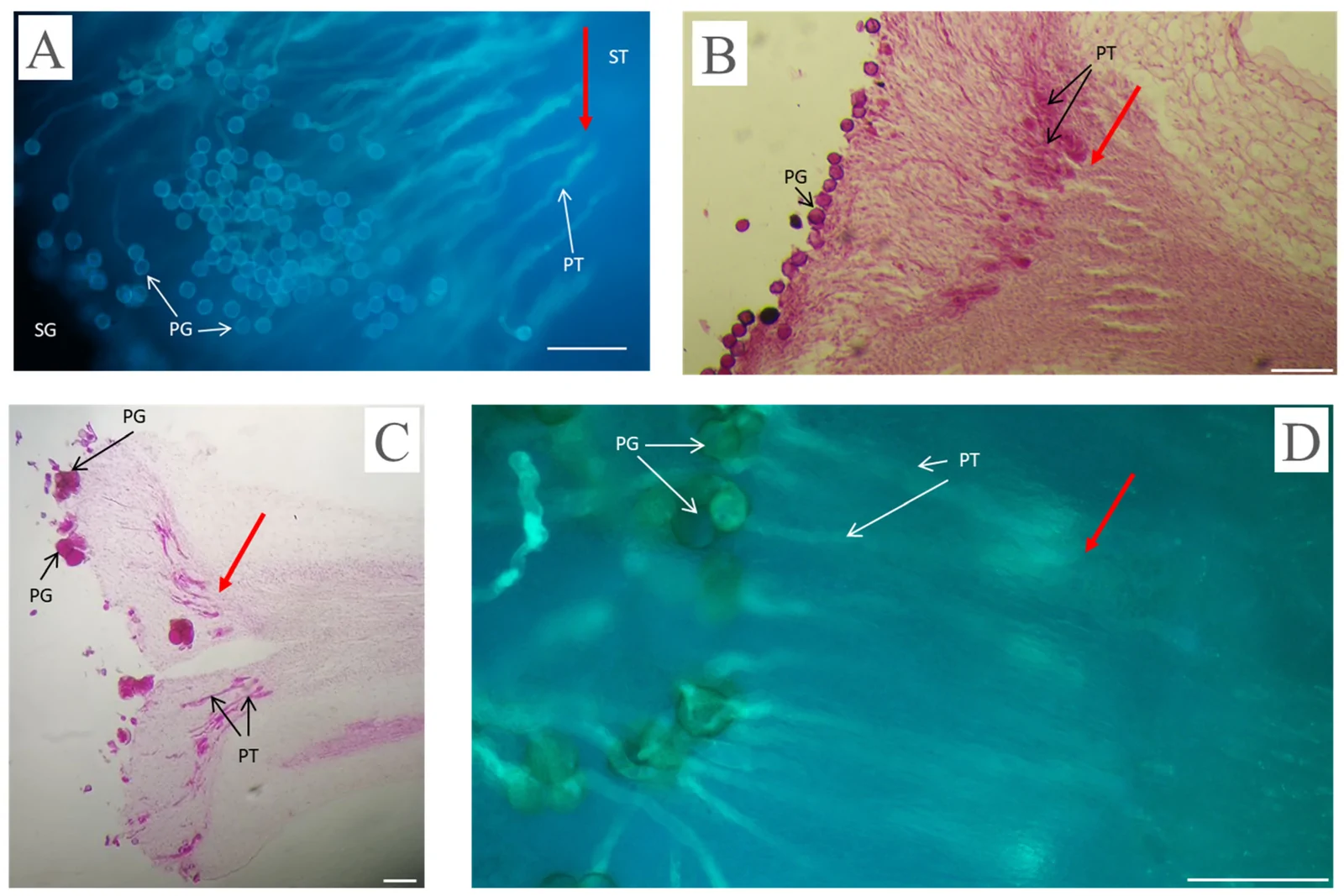
In vivo growth of pollen tubes (PTs) in pistil tissues during intergeneric hybridization (24 h after pollination). (**A**) Self-compatible *P. hybrida* ♀ × *N. tabacum*. Fluorescence microscopy, aniline blue staining. (**B**) Self-compatible *P. hybrida* ♀ × *N. tabacum*. Feulgen staining (bright-field microscopy). (**C**) Self-compatible *P. hybrida* ♀ × *S. sinuata* ♂. Feulgen staining. (**D**) Self-incompatible *P. hybrida* ♀ × *S. sinuata* ♂. Fluorescence microscopy, aniline blue staining. In all crossing variants, pollen tube growth ceases in the upper part of the style. The point of arrest is marked by a red arrow. SG, stigma; ST, style; PG, pollen grain; PT, pollen tube. Bar = 100 µm.

**Figure 2:**
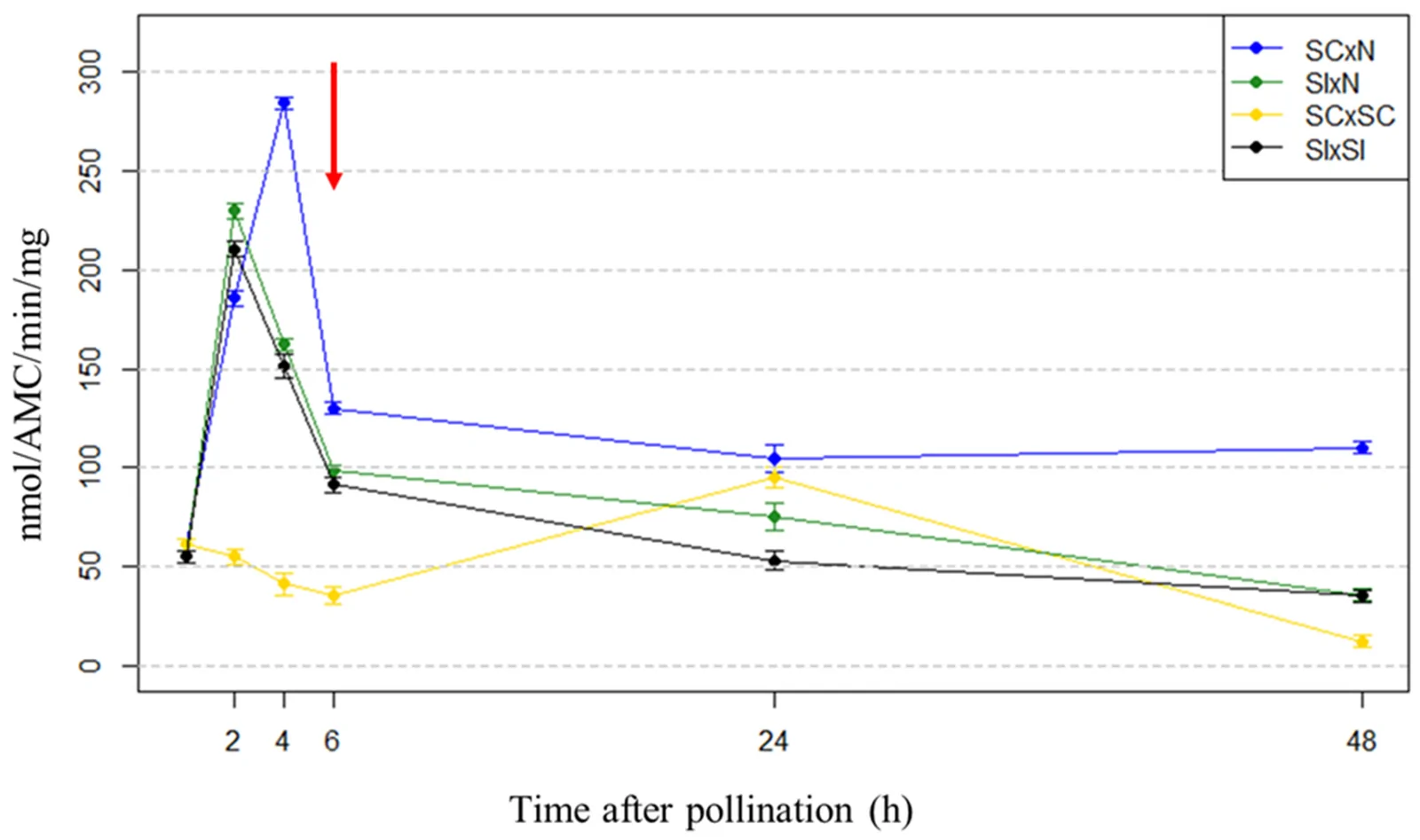
Spectrophotometric assessment of caspase-3-like protease activity in the pollen–pistil system in different pollination variants. Total protein was extracted from 10 pistils without ovaries (~100 mg) and incubated with the caspase-3/DEVDase substrate Ac-DEVD-AMC. Data are presented as the mean rate of peptide cleavage activity (nmol AMC/min/mg). Error bars represent the standard error of the mean (SEM). The arrow indicates the moment of PT growth inhibition caused by self-incompatibility or intergeneric reproductive barriers. Cross combinations: self-compatible *P. hybrida* ♀ × self-compatible *P. hybrida* ♂ (compatible pollination), SC × SC; self-incompatible *P. hybrida* ♀ × self-incompatible *P. hybrida* ♂ (self-incompatible pollination), SI × SI; self-compatible *P. hybrida* ♀ × *N. tabacum* ♂ (intergeneric hybridization), SC × N; self-incompatible *P. hybrida* ♀ × *N. tabacum* ♂ (intergeneric hybridization), SI × N.

**Figure 3:**
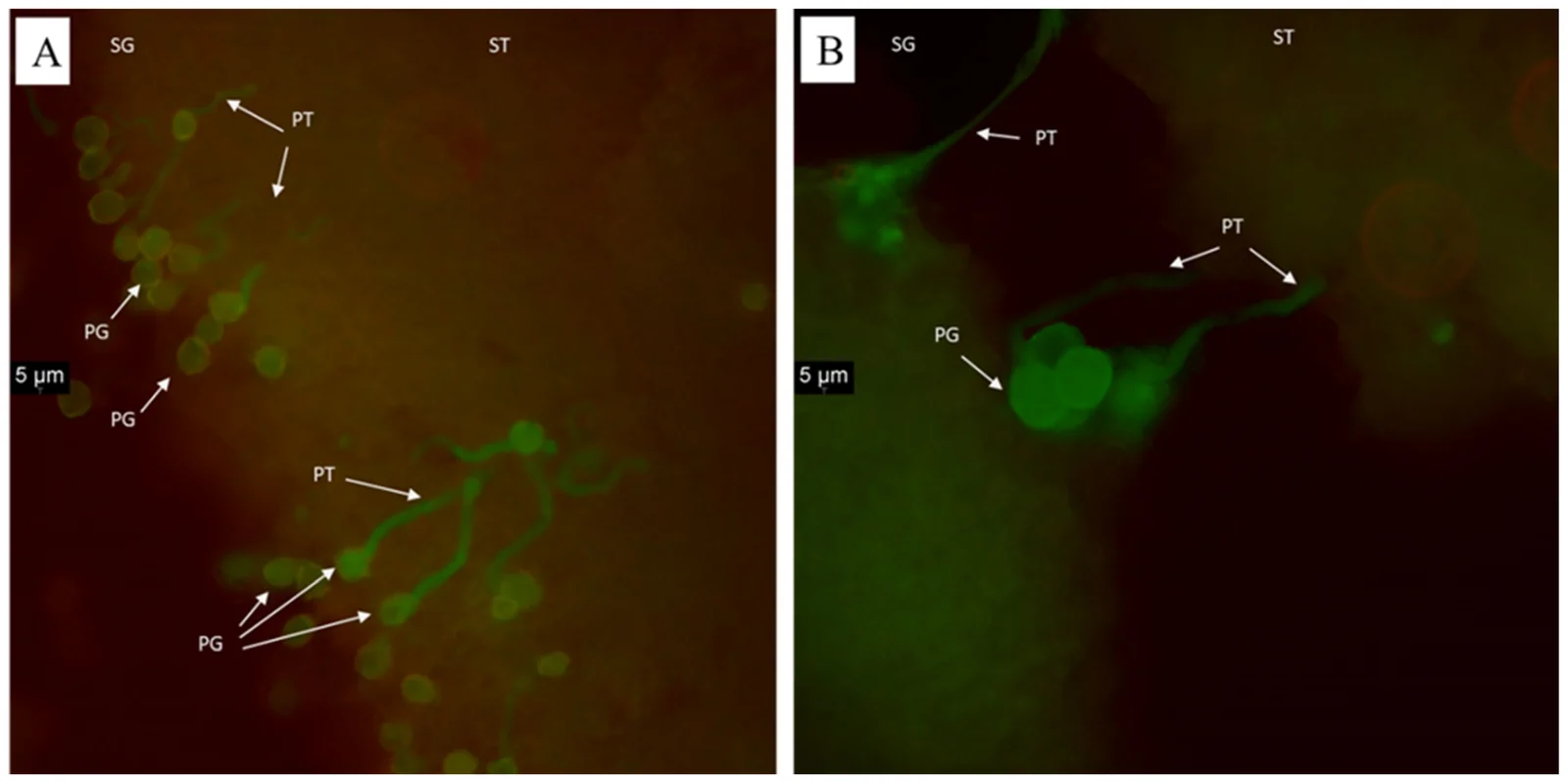
CLP activity in in vivo growing PTs (2 h after pollination). The green fluorescent signal in PGs and PTs indicates the presence of CLP activity in these cells. (**A**) Self-compatible *P. hybrida* ♀ × *N. tabacum* ♂ (intergeneric hybridization), SC × N. (**B**) Self-compatible *P. hybrida* ♀ × *S. sinuata* ♂ (intergeneric hybridization), SC × SS. SG, stigma; ST, style; PG, pollen grain; PT, pollen tube.

**Table 1:** Effect of petunia stigma treatment with the caspase-3 inhibitor (Ac-DEVD-CHO, 0.25 mM) on in vivo pollen tube growth during intergeneric hybridization (µm).

Time After Pollination	Type of Hybridization							
	Distant Hybridization				Distant Hybridization with an Inhibitor			
	SC × N	SI × N	SC × SS	SI × SS	SC × N	SI × N	SC × SS	SI × SS
2 h	403 ± 13	165 ± 17	303 ± 21	175 ± 11	829 ± 11 ***	468 ± 36 *	596 ± 66 **	532 ± 36 **
4 h	688 ± 88	206 ± 28	484 ± 36	257 ± 27	1141 ± 31 ***	515 ± 5 *	951 ± 35 ***	961 ± 73 ***
6 h	920 ± 72	288 ± 33	765 ± 89	319 ± 33	1238 ± 91 ***	1077 ± 132 ***	1114 ± 66 ***	1068 ± 36 ***
24 h	1005 ± 48	388 ± 36	962 ± 78	318 ± 17	1431 ± 13 ***	1423 ± 41 ***	1216 ± 55 **	1069 ± 34 ***
48 h	1049 ± 48	379 ± 16	1000 ± 27	323 ± 11	-			

Data are presented as mean ± standard error of the mean (SEM; n = 3–5). For each time interval, a two-way ANOVA was performed with the factors “inhibitor treatment” (control/0.25 mM) and “cross combination” (SC × N, SI × N, SC × SS, SI × SS). Asterisks denote significant differences between inhibitor-treated variants and the corresponding untreated controls at the same time point: * *p* < 0.05; ** *p* < 0.01; *** *p* < 0.001 (Tukey’s post hoc test). Dash (-) indicates no data available.

**Table 2:** Comparison of self-incompatibility (SI) and intergeneric reproductive barrier (IRB)-induced pollen tube rejection in Solanaceae.

Feature	Self-Incompatibility Solanaceae Type	Intergeneric Reproductive Barrier
Biological purpose	Prevents self-fertilization; promotes outcrossing [29]	Prevents hybridization between different species/genera [56]
Genetic control	S-locus (S-RNase + SLF/SFBB genes) [33,34,35,36]	Largely unknown; likely polygenic [52,53,54]
Pollen tube death mechanism	Programmed cell death (PCD) [17,19]	Programmed cell death (PCD) [this study]
CLP activation	Sharp increase at 2–4 h after pollination [47]	Sharp increase at 2–4 h after pollination [this study]
Localization of CLP activity	Within pollen tubes [47]	Within pollen tubes (Figure 3)
Timing of growth arrest	6–8 h after pollination [17,50,60]	6–8 h after pollination [this study], [50,60]
Effect of caspase-3 inhibitor (Ac-DEVD-CHO)	Complete rescue, seed set achieved [17]	Partial rescue, no seed set achieved (Table 1)
Upstream signaling	S-RNase-mediated RNA degradation [37,38]	Unknown; likely involves multiple signals
Evolutionary role	Maintains genetic diversity within species [27,30]	Maintains reproductive isolation between species [25,55]

## Data Availability

The original contributions presented in this study are included in the article. Further inquiries can be directed to the corresponding author.

## Abbreviations

The following abbreviations are used in this manuscript:

IRB	Intergeneric reproductive barrier
CLPs	Caspase-like proteases
DEVDase	Asp-Glu-Val-Asp-ase
Ac-DEVD-CHO	N-acetyl-Asp-Glu-Val-Asp-al
PG	Pollen grain
PT	Pollen tube
SI	Self-incompatibility
SC	Self-compatible
UI	Unilateral incompatibility
PCD	Programmed cell death
